# Estimated pulse wave velocity as a measure of vascular aging

**DOI:** 10.1371/journal.pone.0280896

**Published:** 2023-01-26

**Authors:** Kevin S. Heffernan, Lee Stoner, Andrew S. London, Jacqueline A. Augustine, Wesley K. Lefferts

**Affiliations:** 1 Department of Exercise Science, Syracuse University, Syracuse, New York, United States of America; 2 Department of Exercise and Sport Science, University of North Carolina, Chapel Hill, North Carolina, United States of America; 3 Department of Sociology, Maxwell School of Citizenship & Public Affairs, Syracuse University, Syracuse, New York, United States of America; 4 Department of Kinesiology, SUNY Cortland, Cortland, New York, United States of America; 5 Department of Kinesiology, Iowa State University, Ames, Iowa, United States of America; University of Perugia, ITALY

## Abstract

**Background:**

Carotid-femoral pulse wave velocity (cfPWV), the referent measure of aortic stiffness, is an established measure of vascular aging. In studies where cfPWV cannot be measured, alternative methods are needed to help promote research on vascular aging. This study examines the construct validity of a measure of PWV estimated from age and blood pressure (ePWV). The specific aims of the study are to: 1) explore the strength of association between ePWV, cfPWV, and other established measures of vascular aging; 2) examine the sensitivity and specificity of elevated ePWV (≥10m/s) in relation to elevated cfPWV (≥10m/s).

**Methods:**

We measured cfPWV in two-hundred and fifty-two adults (mean age 57±12 years, 48% female) and calculated each participant’s ePWV from their age and brachial blood pressure. Additional measures of vascular aging included: carotid intima-media thickness (cIMT); carotid stiffness measured as elastic modulus (cEp); and carotid augmentation index (cAIx).

**Results:**

The correlations between cfPWV and measures of vascular aging were: cEp (r = 0.36), cIMT (r = 0.49), and cAIx (r = 0.04). The correlations between ePWV and measures of vascular aging were: cEp (r = 0.45), cIMT (r = 0.60), and cAIx (r = 0.24). The correlation between ePWV and cfPWV was (r = 0.67). The sensitivity and specificity of elevated ePWV (≥ 10 m/s) for concomitantly identifying high cfPWV (≥ 10 m/s) were 85.4% and 73.0% respectively.

**Conclusion:**

ePWV is associated with established measures of vascular aging, such as carotid thickness, carotid stiffness and carotid augmentation index. ePWV may be a useful tool to help promote research on vascular aging.

## Introduction

A central tenet of geroscience is that physiological aging can be influenced by contextual and behavioral changes, as well as lifestyle and medical interventions, that enhance healthspan (i.e., the compression of morbidity until the end of life) [1, 2]. Within the field of geroscience, vascular aging is emerging as an important health construct both because it is malleable and because it is associated with a broad array of health outcomes in mid- and later life. While healthy vascular aging is associated with independence, improved quality of life, and longevity [3, 4], early and accelerated vascular aging is associated with the emergence of various chronic diseases, disability, frailty, and premature death [5]. Physiological aging, which may or may not coincide precisely with chronological age, instigates structural and functional changes to the vasculature that result in a range of physiological mal-adaptations, including loss of vascular reactivity, increased vascular thickness, and increased vascular stiffness [6, 7]. Although there are many methods available to assess vascular aging, aortic stiffness assessed by carotid-femoral pulse wave velocity (cfPWV) is considered the referent measure [8, 9]. Increased cfPWV (i.e., increased vascular aging) causes target organ damage and, in well-controlled statistical models, is an independent predictor of physical decline, cognitive decline, morbidity, and mortality [8].

Although cfPWV is a robust measure of vascular aging, it is not available in many research studies for a variety of reasons, including financial constraints, lack of access to the specialized equipment needed to measure it, and the absence of trained personnel. In research studies in which cfPWV cannot be measured, low-cost and easily implemented alternatives are needed to help advance research on vascular aging and its consequences. Recently, equations for estimating pulse wave velocity (PWV) using chronological age and blood pressure as inputs have been published [10, 11]. Research has documented that estimated pulse wave velocity (ePWV) is: correlated with cfPWV, with correlations ranging between 0.5 and 0.7; able to predict diseases of vascular aging, such as atrial fibrillation, stroke, left-ventricular hypertrophy, heart failure, and cognitive dysfunction; and associated independently with cardiovascular disease (CVD) and all-cause mortality after traditional CVD risk factors, age, and blood pressure are controlled statistically [12–18]. ePWV has also been shown to predict CVD events following adjustment for cfPWV [19], suggesting that ePWV may be capturing other aspects of vascular aging distinct from those captured by cfPWV. However, to date, this has not been examined.

This current study examines the construct validity of ePWV as a measure of vascular aging. We first compare the strength of the associations between ePWV, cfPWV, and three established measures of vascular aging: carotid intima-media thickness (cIMT); carotid stiffness, measured as the elastic modulus (cEp); and the carotid augmentation index (cAIx). Next, using a previously established cut-point, we assess the sensitivity and specificity of elevated ePWV for identifying elevated cfPWV. Together, these findings advance geroscience and research on vascular aging by providing evidence related to the construct validity of ePWV in relation to the gold-standard measure (cfPWV) and other measures of vascular aging. Documentation of moderate to high construct validity provides an evidence base that supports the use of ePWV in research studies in which age and blood pressure are measured, but cfPWV and other measures of vascular aging are not.

## Materials and methods

We collected data from 252 participants between 2013 and 2018 in the Human Performance Laboratory at Syracuse University [20]. Although we compiled data from three separate studies, we collected all data in a uniform and systematic fashion following published international consensus guidelines.

### Participants

For all three of the distinct studies, we recruited generally healthy volunteers varying in CVD risk factor burden from the local Syracuse community and Syracuse University by means of radio and newspaper advertisement, e-mail listservs, social media posts, and posted flyers. We required participants to be non-smokers and, based on self-report, to have no history of dementia, depression, stroke, previous cardiovascular events, diabetes mellitus, renal disease, pulmonary disease, neurological disease, or recent head trauma (e.g., concussion) as determined by a primary health care provider (i.e., “Has your primary healthcare provider ever informed you that you have…”).

### Ethics statement

All study procedures were approved by the Syracuse University Institutional Review Board. All participants gave written informed consent prior to data collection.

### Study design

Participants were instructed to refrain from caffeine, non-essential medication (e.g., nutritional supplements), alcohol, and physical exercise on the day they came to the lab for the vascular assessments. All visits to the lab were scheduled in the morning. We tested pre- and peri-menopausal women during the early follicular phase of their menstrual cycle, with no standardization for post-menopausal women. We assessed participants taking oral contraceptives during the placebo (i.e., inactive pill) week. We initiated all vascular assessments after participants rested quietly in the supine position for 10 minutes.

### Descriptive characteristics

As described previously [20], we assessed anthropometrics (i.e., weight, height) via electronic scale and stadiometer in order to calculate body mass index (BMI). Percent body fat was estimated via air displacement plethysmography (Bod Pod, Cosmed, Concord CA). Fasting plasma glucose and serum lipoproteins (i.e., total cholesterol, low- and high-density lipoproteins) were determined via finger stick blood sample (Cholestech, Alere Medical) after an overnight fast and abstinence from alcohol, caffeine, and physical exercise.

### Vascular measures

#### Estimated pulse wave velocity (ePWV)

Estimated pulse wave velocity (ePWV) was derived for individual participants based on their study-assessed CVD risk factor status using one of the two algorithms published by Greve et al. [10]:

1. No cardiovascular disease risk factor:
  4.62–0.13 * age + 0.0018 * age^2^ + 0.0006 * age * mean arterial pressure (MAP) + 0.0284 * MAP
2. ≥1 cardiovascular disease risk factor:
  9.587–0.402 * age + 4.560 * 10^−3^ * age2–2.621 * 10^−5^ * age^2^ * MAP + 3.176 * 10^−3^ * age * MAP—1.832 * 10^−2^ * MAP

In these equations, we entered self-reported age in years and mean arterial pressure (MAP), which was calculated as diastolic blood pressure (DBP) + 0.4*(systolic blood pressure [SBP]-DBP). Brachial SBP and DBP were measured as the average of at least 2 measurements within 5 mmHg via an oscillometric blood pressure cuff as described previously [20]. We selected the ePWV equation based on the presence or absence of CVD risk factors, including: age (males ≥45 years, females ≥55 years); BMI (≥30 kg/m^2^); hypertension diagnosis; dyslipidemia (low-density lipoprotein ≥130 mg/dl, high-density lipoprotein <40 mg/d); total cholesterol >200 mg/dl); glucose (≥100 mg/dl); and self-reported use of medications to treat hypertension or dyslipidemia. Note that multipliers in equation 2 have been truncated to 3 decimal places for brevity.

#### Aortic stiffness

We assessed cfPWV, the referent measure of aortic stiffness and vascular aging, with applanation tonometry [8]. We captured ten seconds of carotid and femoral artery blood pressure waveforms and simultaneous electrocardiogram (ECG) in order to derive cfPWV (AtCor Medical, Sydney, Australia). We determined cfPWV by computing D/Δt, where D is the transit distance between carotid and femoral pulse sites and Δt is the time delay from the peak ECG R-wave to the foot of the corresponding pressure waveform between the carotid and femoral waveforms. Transit distance between sites was determined as (suprasternal notch—femoral distance)–(suprasternal notch—carotid distance), which is consistent with cfPWV assessment recommendations [8].

#### Carotid intima-media thickness

We assessed carotid intima-media thickness (cIMT) via ultrasound (7.5–10.0 MHz linear-array probe; ProSound α7, Aloka, Tokyo, Japan) using a longitudinal view of the common carotid artery far wall during diastole, which we determined from simultaneous ECG gating. cIMT was assessed via semi-automated digital calipers across a 5 mm region of interest just upstream of the carotid bulb. cIMT was measured as the distance from the lumen-intima interface to the media-adventitia interface.

#### Carotid stiffness and augmentation index

We assessed stiffness of the carotid artery via ultrasound with wall-tracking software (eTracking). Our approach consists of *simultaneously* measuring brachial blood pressure with a cuff (described above), carotid distension waveforms on the left common carotid artery with ultrasound and carotid pressure waveforms on the right common carotid artery with applanation tonometry. This is described next in more detail. Distances between the near and far wall lumen-intima interface are continuously traced via eTracking software to create a distension waveform akin to a pressure waveform [21]. At least five carotid distension waveforms from a 10–12 second epoch are ensemble averaged to generate a representative waveform. To calibrate the carotid distension waveform, inbuilt software for the Aloka platform prompts entering systolic and diastolic pressures, with no option to calibrate the waveform using mean pressure. As brachial and carotid systolic pressures can differ substantially across the life course owing to changes in pressure amplification, we chose to calibrate the carotid distension waveform using carotid systolic rather than brachial systolic pressures [22]. Carotid pressure waveforms were obtained by simultaneous applanation tonometry on the contralateral common carotid artery. These carotid waveforms were saved and then calibrated using brachial mean and diastolic pressure, assuming that mean and diastolic pressure remain more constant throughout the vascular tree, from simultaneous brachial cuff measures [23]. Carotid stiffness was calculated using carotid pressures from simultaneous carotid tonometry, calibrated against brachial mean arterial and diastolic pressure, as a local carotid elastic modulus: cEp = (systolic pressure–diastolic pressure) / ([systolic diameter–diastolic diameter] / diastolic diameter). Carotid pressure waveforms were also used to measure the augmentation index (cAIx) calculated as [(P2—P1) / pulse pressure*100], where P1 and P2 are the early- and late-systolic peaks of the pressure waveform, respectively, and taken as a measure of global pressure from wave reflections.

### Statistical analyses

After describing the characteristics of the sample, we examine the strength of the associations between cfPWV, ePWV, age, MAP, and other measures of vascular aging in order to preliminarily assess construct validity. Our aim is to estimate how well the gold standard cfPWV and the ePWV measures correlate with other direct measures of vascular aging: vascular stiffness, thickness, and pressure from wave reflections. We estimated Pearson moment correlation coefficients (r). Although there is no universal criterion for strength of associations, in general, r values of <0.2, 0.2–0.4, 0.4–0.70, 0.70–0.9 and >0.9 indicate negligible, weak, moderate, strong, and very strong correlation, respectively [24].

**Fig 1:**
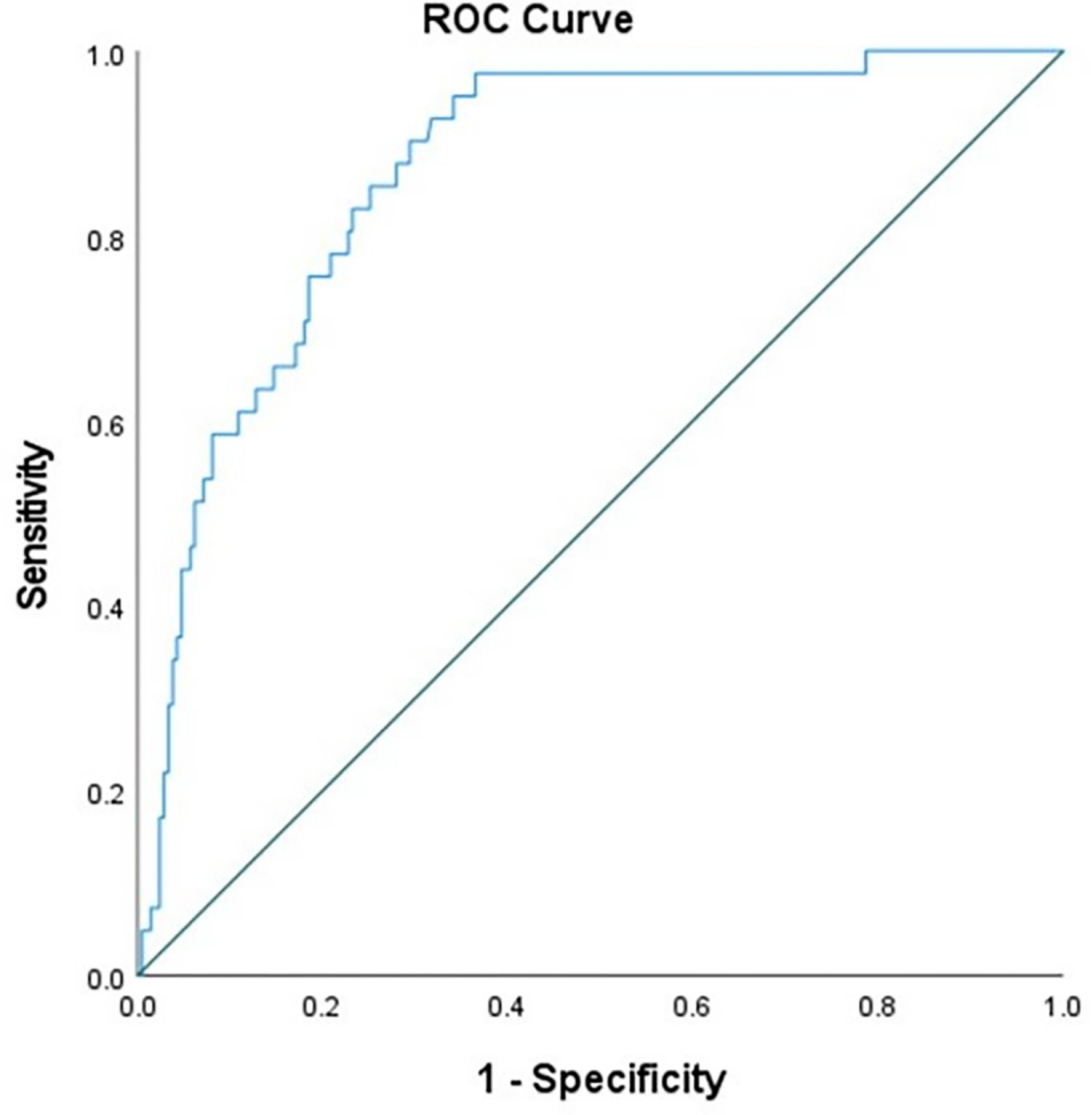
Receiver operator characteristic (ROC) curve for ePWV as a predictor of higher cfPWV (defined as a value > 10.0 m/s).

Next, we directly estimate the sensitivity, specificity, negative predictive value, positive predictive value, and accuracy for identifying elevated cfPWV. We grouped participants into low and high PWV based on a previously established cut-point of cfPWV ≥10m/s [25, 26]. Prior to this, we confirmed use of this cut-point for ePWV within our dataset by generating a receiver operator characteristic (ROC) curve (Fig 1) and assessing the Youden Index (J statistic, defined as the maximum value obtained for sensitivity + specificity -1). The area under the curve (AUC) for ePWV entered as a continuous variable for discriminating high and low cfPWV as a dichotomous variable was 0.87 (Fig 1, p<0.05) and a cut point for ePWV of 10.0 m/s was confirmed as offering high diagnostic discrimination for identifying a concomitant high cfPWV (defined as ≥10m/s). Participants were then categorized as True Negative (ePWV and cfPWV <10m/s), True Positive (ePWV and cfPWV ≥10m/s), False Negative (ePWV <10m/s, cfPWV ≥10m/s) and False Positive (ePWV ≥10m/s, cfPWV <10m/s). We then calculated sensitivity, specificity, negative predictive value (NPV), positive predictive value (PPV) and accuracy as described in Table 1.

**Table 1:** Definitions of sensitivity, specificity, Negative Predictive Value (NPV), Positive Predictive Value (PPV) and accuracy.

	cfPWV ≥10m/s	cfPWV <10m/s	
ePWV ≥10m/s	True Positive (TP)	False Positive (FP)	PPV = $\frac{TP}{\left(TP+FP\right)}$
ePWV <10m/s	False Negative (FN)	True Negative TN	NPV = $\frac{TN}{\left(FN+TN\right)}$
	Sensitivity = $\frac{TP}{\left(TP+FN\right)}$	Specificity = $\frac{TN}{\left(FP+TN\right)}$	Accuracy = $\frac{TP+TN}{\left(TP+TN+FP+FN\right)}$

As described in greater detail below, we identified very few False Negative cases and a relatively high proportion of False Positive cases. Thus, we conducted a secondary analysis to describe the characteristics and vascular aging parameters of three groups—True Negative, True Positive, False Positive—to better understand factors that might contribute to misclassification of cfPWV by ePWV. This was done using one-way analysis of variance (ANOVA) with Bonferonni-adjusted post-hoc comparisons for continuous variables and Chi-Square tests for categorical variables.

Finally, we conducted an additional secondary analysis to help us better understand the association between ePWV and cfPWV. Specifically, we examined the association between ePWV and cfPWV in a multivariate context to determine the extent to which ePWV predicts cfPWV once other factors are taken into account. Using ordinary least squares (OLS) regression, we regressed ePWV on cfPWV with controls for age, SBP, DBP, and other factors known to affect vascular aging and the association of age with blood pressure across the lifespan: sex, body fat, total cholesterol to HDL ratio, glucose, and self-reported diagnoses of hypertension, dyslipidemia, and statin use. Goodness of fit for nested models was assessed using the change in R^2^.

All analyses are unweighted. We set statistical significance a priori at p<0.05. We conducted all analyses using the Statistical Package for the Social Sciences (SPSS, Version 27, IBM, Chicago IL).

## Results

Table 2 presents a description of the sample, including mean values (± the standard deviation [SD]) for traditional CVD risk factors and measures of vascular aging. Participants were between the ages of 35 and 85 years. Approximately one-quarter were normal weight (BMI < 25 kg/m^2^), 49% were overweight (BMI ≥ 25 but < 30 kg/m^2^), and 23% were obese (BMI ≥ 30 kg/m^2^). Overall, 18% were using statins and 27% were using anti-hypertensives. Participants, on average, had two CVD risk factors (range = 0–5); 77% had at least one CVD risk factor.

**Table 2:** Descriptive characteristics and traditional CVD risk factors.

Variable	Mean ± SD
Age, years	57±12
Female Sex, n(%)	122(48.4)
Body mass index, kg/m^2^	27.1±4.3
Body fat, %	29.0±10.3
Systolic blood pressure, mmHg	124±13
Diastolic blood pressure, mmHg	78±8
Hypertension, n(%)	69(27.4)
Dyslipidemia, n(%)	90(35.7)
Fasting Blood Chemistries	
Total cholesterol, mg/dl (*n* = 244)	192±36
LDL cholesterol, mg/dl (*n* = 214)	110±32
HDL cholesterol, mg/dl (*n* = 245)	60±19
Triglycerides, mg/dl (*n* = 235)	109±62
Glucose, mg/dl (*n* = 244)	93±12

(*n = 252* unless indicated otherwise)

### Correlation between ePWV and measures of vascular aging

Table 3 presents the correlations between age, MAP, ePWV, cfPWV, cIMT, cEp and cAIx. As shown in Table 3, each of the variables is significantly correlated with all of the others, although the magnitude of the associations varies. Unsurprisingly, ePWV is strongly associated with age and moderately associated with MAP, which are its constituent components. Notably, the strength of the associations between ePWV and other measures of vascular aging is greater than the strength of the associations between cfPWV and those measures. The association between ePWV and cfPWV would be viewed as moderately-strong based on our a priori criteria.

**Table 3:** Inter-associations across ePWV and vascular aging measures.

Variable^a^	Age	MAP	ePWV	cfPWV	cIMT	cEp
MAP	0.23***					
ePWV	0.93***	0.53***				
cfPWV	0.61***	0.35***	0.67***			
cIMT^b^	0.54***	0.23***	0.60***	0.49**		
cEp^b^	0.38***	0.41***	0.45***	0.36**	0.26***	
cAIx^c^	0.20**	0.24***	0.24***	0.04	0.15*	0.15*

Significance: *** = p<0.001; ** = p<0.01; * = p<0.05

Table Notes

a. ePWV, estimated pulse wave velocity; cfPWV, carotid-femoral pulse wave velocity; cIMT, carotid intima-media thickness; cEp, carotid elastic modulus; Ao AIx, aortic augmentation index; Ao Pb, aortic wave reflection pressure; Ao AIx75, aortic augmentation index.

b. n = 251

c. n = 250

### Relationship between ePWV and cfPWV

**Fig 2:**
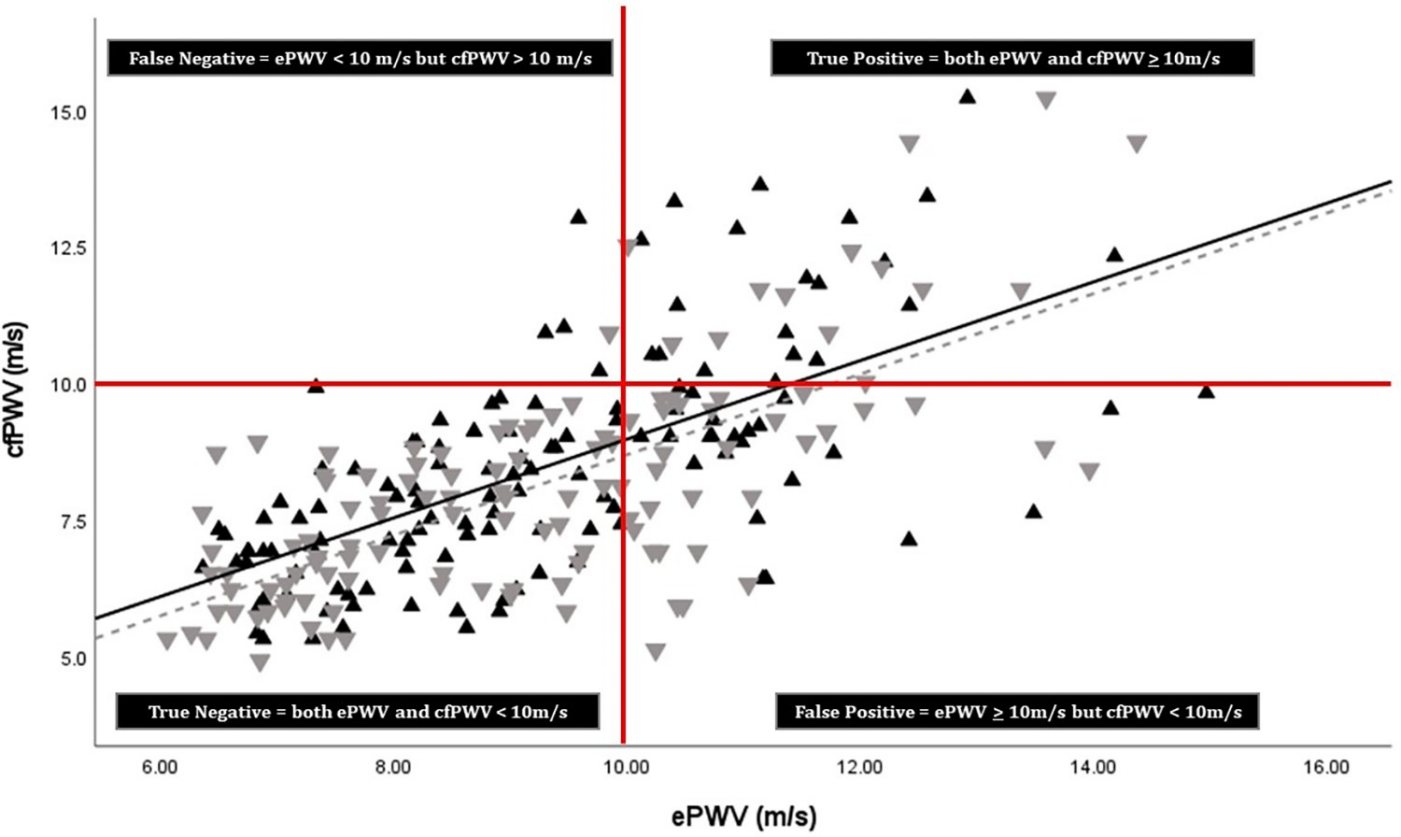
Scatter-plot showing correlation between ePWV and cfPWV for men (grey triangles) and women (black triangles). The red grid overlaying the scatterplot demarcates True and False Negatives and Positive cases, respectively, using 10.0 m/s as the cut-point for both ePWV and cfPWV.

Fig 2 presents a scatter-plot with ePWV on the x-axis and cfPWV on the y-axis for men (black triangles) and women (gray triangles). The red grid overlaying the scatterplot demarcates True and False Negatives and Positive cases, respectively, using 10.00 as the cut-point for both ePWV and cfPWV (see Statistical Analyses section above for the details on how the cut-point was determined). Overall, 16.3% (n = 41) scored Positive for elevated cfPWV (> 10 m/s). As seen in Fig 2, the weight of the scatter falls in the True Negative quadrant; with ePWV performing relatively well, identifying True Negative cases (n = 154). Only 6 participants fall in the False Negative quadrant, while 35 participants fall in the True Positive quadrant (positive on both measures) and 57 participants fall in the False Positive quadrant (positive on the ePWV measure only).

### Sensitivity and specificity of ePWV for correctly identifying high cfPWV

Table 4 displays sensitivity, specificity, negative predictive value, positive predictive value, and accuracy for ePWV in identifying high cfPWV. Overall, ePWV demonstrated high sensitivity and moderate specificity. Notably, if we use equation 2 above to calculate ePWV for all participants rather than just those with one or more CVD risk factor, sensitivity and negative predictive value would increase to 100%.

**Table 4:** Sensitivity, specificity, Negative Predictive Value (NPV), Positive Predictive Value (PPV) and accuracy.

	Individualized ePWV^a^	Standardized ePWV^b^
Sensitivity	85.4%	100.0%
Specificity	73.0%	73.0%
NPV	96.3%	100.0%
PPV	38.0%	44.1%
Accuracy	75.0%	77.7%

Table Notes

a. Individualized ePWV, using equation 1 and equation 2 based on individual participant CVD risk factors (see Methods section for details).

b. Standardized ePWV, assuming moderate CVD risk and using equation 2 for all participants (see Methods section for details).

### Secondary analyses

We conducted secondary analyses to help us understand the potential value of ePWV as a measure of vascular aging. The first aimed to better understand the characteristics of the relatively large group of participants falling into the False Positive category, which lowered the specificity and positive predictive value of ePWV. Table 5 displays selected characteristics across three groups based on both ePWV and cfPWV classifications; we exclude the False Negative group given the small sample size (n = 6). In general, participants classified in the True Negative group were younger, on average, with lower traditional CVD risk factor burden compared to other groups. Specifically, the True Negative group had lower ePWV, cfPWV, cIMT, cEp, and cAIx (p<0.05 for all). In contrast, the True Positive group had higher cfPWV, cIMT, cEp and cAIx compared to the True Negative group (p<0.05 for all). Interestingly, aside from differences in cfPWV, there were no differences in measures of traditional CVD risk or vascular aging measures (cIMT, cEp and cAIx) between True Positive and False Positive groups (p>0.05). That is, the True Positive and False Positive groups had comparable and higher CVD risk (i.e., CVD risk factors and vascular aging measures) compared to the True Negative group.

**Table 5:** Descriptive and vascular characteristics of participants according to both ePWV and cfPWV strata.

Variable	ePWV <10m/s	ePWV ≥10m/s	ePWV≥10m/s	p-value
	cfPWV <10m/s	cfPWV≥10m/s	cfPWV<10m/s	
	(True Negative)	(True Positive)	(False Positive)	
	n = 154	n = 35	n = 57	
Age, years	49±9	71±6^†^	67±6^†^	<0.001
MAP, mmHg	93±8	101±9^†^	102±8^†^	<0.001
Female Sex, n(%)	76(49.4)	14(40.0)	31(54.5)	0.41
Body mass index, kg/m^2^	27.1±4.4	27.5±4.9	26.9±3.6	0.83
Body fat, %	27.9±9.7	32.0±11.0	29.6±10.8	0.08
Hypertension, n(%)	24(15.6)	17(48.6)^†^	26(45.6)^†^	<0.001
Dyslipidemia, n(%)	35(22.7	21(60.0)^†^	32(56.1)^†^	<0.001
Total cholesterol, mg/dl^a^	193±34	183±39	193±39	0.31
LDL cholesterol, mg/dl^b^	112±32	98±33	109±33	0.08
HDL cholesterol, mg/dl^c^	61±18	56±18	61±22	0.37
Triglycerides, mg/dl^d^	97±56	142±75^†^	118±65	<0.001
Glucose, mg/dl^a^	93±12	93±14	95±11	0.31
ePWV, m/s	8.0±1.0	11.6±1.1^†^	11.0±1.1^†‡^	<0.001
cfPWV, m/s	7.3±1.2	11.9±1.5^†^	8.4±1.2^†‡^	<0.001
Carotid IMT, mm^e^	0.56±0.12	0.73±0.12^†^	0.69±0.12^†^	<0.001
Carotid Ep, kPa^e^	81.8±29.5	113.1±52.8^†^	113.5±52.0^†^	<0.001
Carotid AIx, %	16±17	19±14	24±17^†^	0.009

a. Significance: †Significantly different from true negative (reference), p<0.05; ‡Significantly different from true positive, p<0.05

Table Notes

a. True Negative, n = 151; True Positive, n = 32; False Positive, n = 55

b. True Negative, n = 131; True Positive, n = 32; False Positive, n = 45

c. True Negative, n = 153; True Positive, n = 32; False Positive, n = 54

d. True Negative, n = 144; True Positive, n = 32; False Positive, n = 53

e. True Negative, n = 154; True Positive, n = 35; False Positive, n = 56

Secondary analyses were also conducted to better understand the association of ePWV and cfPWV independent of other known correlates of vascular aging. To do this, we used a series of multiple linear regression models to explore whether ePWV predicts cfPWV net of other correlates of cfPWV. As shown in Table 6, we began with a model that included only age and MAP, the factors that are used to estimate PWV. The results indicated that age (standardized B = 0.6, p < 0.001) and MAP (standardized β = 0.2, p < 0.001) accounted for 43.5% of the variance in cfPWV. When the set of traditional CVD risk factors were added to the model, 45.5% of the variance in cfPWV was explained. In the final stage, we added ePWV to the model and found that it explained an additional, statistically significant 1.9% of the variance in cfPWV (standardized β = 0.78, p = 0.005). Thus, ePWV appears to be capturing information on cfPWV beyond just age, MAP, and other known correlates of vascular aging. In an unadjusted model, ePWV was associated with cfPWV (standardized B = 0.7,), explaining 45.1% of the variance in cfPWV (p<0.05). Moreover, there was no sex-by-ePWV interaction (p = 0.46) and repeating the analyses in sex-specific models yielded results that are not appreciably different than those reported.

**Table 6:** Multiple regression model with cfPWV as the dependent variable.

	Model 1		Model 2		Model 3	
Variable^a^	Unstandardized β (SE)	95% CI	Unstandardized β (SE)	95% CI	Unstandardized β (SE)	95% CI
Age	0.10 (0.01)	**0.08–0.11**	0.08 (0.01)	**0.06–0.10**	-0.03 (0.04)	-0.10–0.05
MAP	0.05 (0.01)	**0.03–0.07**	0.04 (0.01)	**0.02–0.07**	-0.01 (0.02)	-0.06–0.03
Female Sex			-0.34 (0.22)	0.78–0.11	-0.39 (0.22)	-0.82–0.05
Body Fat			0.02 (0.01)	**0.01–0.04**	0.03 (0.01)	**0.01–0.05**
Total C/HDL			0.10 (0.08)	-0.06–0.26	0.13 (0.08)	-0.03–0.29
Glucose			0.01 (0.01)	-0.02–0.01	0.01 (0.01)	-0.02–0.01
Hypertension			0.32 (0.25)	-0.17–0.80	0.37 (0.24)	-0.11–0.85
Dyslipidemia			-0.04 (0.26)	-0.55–0.48	0.04 (0.26)	-0.47–0.55
Statins			-0.02 (0.31)	-0.61–0.65	-0.06 (0.31)	-0.68–0.56
ePWV					0.82 (0.29)	**0.26–1.38**

Table Notes

a. MAP, mean arterial pressure; C, cholesterol; HDL, high-density lipoprotein; ePWV, estimated pulse wave velocity; SE, standard error; 95% CI, 95% confidence interval.

## Discussion

This study set out to explore ePWV as a measure of vascular aging when compared to the referent measure cfPWV and other established measures of vascular aging. To this end, this study specifically sought to: examine the strength of association of ePWV with other measures of vascular aging; and assess the sensitivity and specificity of elevated ePWV for discriminating elevated cfPWV. Our results are as follows: 1) ePWV was associated with cfPWV, carotid intima-media thickness, carotid stiffness, and the carotid augmentation index supporting construct validity as a measure of vascular aging; 2) ePWV itself explained < 50% of the variance in cfPWV; and 3) although ePWV performed well when identifying individuals with low cfPWV and moderately well identifying individuals with elevated cfPWV, ePWV misclassified ~20% of the sample as having elevated PWV (> 10 m/s) when these individuals had cfPWV values < 10 m/s (i.e., false positives). Taken together and findings suggest that while ePWV is only moderately associated with cfPWV, ePWV may still be a useful tool to help promote research on vascular aging.

Though cfPWV is considered the referent measure of aortic stiffness and vascular aging, the travelled path from carotid to femoral sites is estimated to be ~60% aorta and ~40% femoral artery [24]. Thus, this measure is sensitive to aging of both central and peripheral arteries. As such, ePWV may also be detecting aspects of central and peripheral vascular aging [27]. Estimated PWV was associated with three other measures of vascular aging in our study: carotid intima-media thickness (cIMT), carotid stiffness (cEp), and carotid augmentation index (cAIx). Notably, the strength of the associations between ePWV and these parameters was greater than the strength of the associations between cfPWV and these parameters. Carotid IMT, carotid stiffness, and carotid AIx each capture a unique aspect of vascular aging complementary to, yet distinct from, cfPWV. Carotid IMT is a marker of CVD risk [28] that has been shown to add incremental value to cfPWV as a predictor of CVD risk [29]. PWV is now largely considered a measure of arteriosclerosis (i.e., an outside-in process related to structural changes in the adventitial and medial layers from calcification, glycation, elastin fragmentation and degradation, and collagen undulation/deposition and cross-linking) [30]. Carotid IMT is thought to capture a combination of smooth muscle hypertrophy from vascular target organ damage and subclinical atherosclerosis (i.e., an inside-out process of fat accumulation within the vessel wall impacting endothelial function) [31]. Thus, Carotid IMT and cfPWV reflect different aspects of the vascular aging process. Similarly, although both the aorta and carotid arteries are considered large elastic arteries, cfPWV and carotid Ep are not redundant measures of vascular stiffness. The aorta and carotid arteries “age” at different rates with accumulation of CVD risk factors [32]. Moreover, carotid stiffness offers additional insight into vascular aging beyond that of cfPWV [33]. The augmentation index, although often conflated with cfPWV as a measure of arterial stiffness, is a measure of pressure from wave reflections sensitive to both central and peripheral vascular aging [34, 35] and an independent predictor of future cardiovascular events and mortality [36]. AIx and cfPWV each differentially associate with target organ damage and cerebrovascular risk [37, 38]. Furthermore, increases in cfPWV and AIx with age following distinct trajectories. While increases in cfPWV with aging are somewhat linear, increase in AIx with aging is curvilinear; a plateau is seen in late life owing to impedance matching and shifts in the central-peripheral stiffness gradient [35]. Finally, carotid IMT, carotid stiffness, and AIx each have separate and unique inflammatory signatures relative to cfPWV [39]. Taken together and our findings suggest that ePWV has construct validity as a measure of vascular aging and may reflect several vascular aging pathways including, but also distinct from, those captured by cfPWV.

We noted a moderately strong linear association between ePWV and cfPWV (*r* = 0.67). This is consistent with other studies noting associations between ePWV and cfPWV ranging between *r* = 0.5 and 0.7 [10, 40, 41], although some studies report lower correlations of r = 0.3 [42]. ePWV alone explained < 50% of the variance in cfPWV. Associations between ePWV and cfPWV remain when controlling for age, blood pressure, and other CVD risk factors and correlates of vascular aging. We further dichotomized ePWV into lower and higher values based on a cut-point of 10 m/s (which was confirmed for use with ePWV in our study with ROC curve analysis) to assess overall accuracy for ePWV categories predicting cfPWV categories. Treating ePWV as a categorical variable revealed good specificity, with low ePWV correctly identifying participants with concomitant low cfPWV in ~85% of cases. If assuming some CVD present in all participants and using a single equation (equation 2 in the methods), specificity was increased to 100%. Sensitivity (and PPV) of ePWV for correctly categorizing high cfPWV was moderate, with 22.6% of cases “miscategorized” as false positive. However, further scrutiny of these results is warranted. Individuals with ePWV ≥ 10 m/s but cfPWV < 10 m/s (False Positive group) exhibited higher carotid IMT, carotid stiffness, and carotid AIx compared to those with both ePWV and cfPWV <10m/s (True Negative group) and comparable carotid IMT, carotid stiffness, and carotid AIx as those with both ePWV and cfPWV ≥10m/s (True Positive group). Although “miscategorized” as a false positive based on cfPWV strata, individuals with ePWV ≥ 10 m/s may not be viewed collectively as experiencing healthy vascular aging. Our findings suggest that individuals with ePWV values ≥10 m/s may have hastened vascular aging compared with individuals with ePWV values < 10 m/s, irrespective of cfPWV status.

ePWV is a mathematical expression of both MAP and age. Since all vascular aging metrics studied herein are highly sensitive to both age and MAP, it is not surprising that these vascular aging measures correlate more strongly with ePWV than with cfPWV. A measure of PWV derived solely from age and blood pressure cannot be expected to capture all individual-level alterations of the arterial system with aging. PWV depends both chronically and acutely on blood pressure. Long-term exposure to elevated blood pressure can lead to structural remodeling and long-term stiffening of the arterial wall (i.e., “passive” stiffening). Conversely, an acute pressor stimulus will alter vessel wall load bearing and functionally increase stiffness (i.e., “active” stiffening). This can be further complicated by the non-linear stress-strain relationship of the vessel wall such that the vessel will become stiffer with increasing distension. Empirical observations of the approximately exponential pressure-diameter relationship of arteries [43], suggests a non-linear (i.e., quadratic with negative concavity) relationship between PWV and blood pressure [44]. The equations to estimate PWV from age and mean BP include non-linear (quadratic) terms and are expected to capture both active and passive arterial stiffening to some extent. However, at the microscopic/individual level, ePWV may only be a gross match for cfPWV at a given chronological age and observed blood pressure [27]. We believe our findings support use of ePWV as a measure of vascular aging at a more macroscopic/population level. Owing to complex changes in the association between age and blood pressure acutely and across the lifespan, ePWV may be capturing different elements of vascular aging that are related to but also distinct from cfPWV. These complex interactions are also likely bi-directional with age-associated increases in blood pressure contributing to vascular damage and remodeling and vascular damage and remodeling affecting blood pressure (via effects on pressure from wave reflections). Therefore, judicious application of ePWV to population and cohort data where age and blood pressure is measured, but cfPWV is not, may help serve as a hypothesis-generating tool to advance the study of vascular aging. We are not proposing a clinical use for ePWV herein. Additional research is needed to explore the clinical merit of ePWV.

### Limitations and additional considerations

Categorization based on ePWV and cfPWV resulted in a small number (n = 6) of False Negative cases. Considering the limited sample size of participants who were classified as False Negative, we did not include this group in our statistical analyses. Participants in this study had some CVD risk factors (including history of dyslipidemia and hypertension). However, they were still a relatively low CVD risk group: none had diabetes; few had cfPWV ≥ 10 m/s; and none had IMT values exceeding 1.2 mm, which suggests that none had clinically relevant carotid stenosis and overt atherosclerotic disease. Thus, more research is needed to examine use of ePWV as a measure of vascular aging among individuals with higher CVD risk, such as individuals with type 2 diabetes. Participants in this study were predominantly non-Hispanic White of European ancestry. More research is needed to explore the utility of ePWV as a measure of vascular aging in other races and ethnicities.

Consistent with the literature, we used two different equations to estimate PWV. For each participant, we selected the estimation equation based on individual-level Framingham CVD risk factor burden, which uses somewhat arbitrary cut-points to define risk. This strategy affects our estimates of PWV across the age range. As an illustrative example, a male with mean BP = 120 mmHg would experience a ~0.13 m/s increase of ePWV until the age of 44 years. At age 45 with the same mean BP, this individual’s ePWV would rather drastically increase by 0.35 m/s as age now becomes a CVD risk factor necessitating use of a different equation. Parenthetically, this may also be a strength and novel use of ePWV. For example, an individual with CVD risk factors could calculate their ePWV using both equations to see how lowering their risk factor burden could theoretically impact their vascular aging trajectory and subsequent CVD risk. Whether this would motivate healthy behavior change is speculative and requires further study.

We operationally defined cfPWV values > 10 m/s as “high” in accordance with published suggestions and based on our previous work noting increased mortality risk with ePWV > 10 m/s [45]. This cut-point may vary based on the method used to estimate path length for cfPWV. Use of cut-points to stratify risk is currently not recommended by AHA [8] and others [26]. As stated by Townsend et al. in the AHA Position Paper on arterial stiffness, “…risk estimation based on fixed thresholds has several limitations, not least of which are the relatively continuous relationship between risk and cfPWV and the failure to consider factors such as transient elevation of MAP, which may confound cfPWV values because of nonlinear stiffness of the aortic wall [8].” Moreover, a single threshold does not consider the prevailing effect of age on PWV. A cfPWV value of 10.1 m/s may convey different prognostic information in a 70-year-old person and in a 30-year-old person.

## Conclusions

Estimated PWV is associated with cfPWV, carotid IMT, carotid stiffness, and carotid AIx. And while ePWV had high sensitivity and moderate specificity for identifying individuals with elevated aortic stiffness (based on also having high cfPWV), ePWV misclassified ~20% of the sample as having high cfPWV when they did not. Other vascular aging measures such as carotid stiffness, IMT and AIx in these misclassified participants (i.e., in the False positive group) appeared to be elevated compared to corresponding values in the True negative group and comparable to those of the True positive group. This pattern of results suggests that ePWV may provide insight into vascular aging even though it is only moderately correlated with cfPWV. ePWV be a useful tool to promote research on vascular aging in research studies in which age and blood pressure are measured but other measures of vascular aging such as cfPWV are not.

## Supporting information

S1 File (XLSX)
